# Efficacy of CU06-1004 via regulation of inflammation and endothelial permeability in LPS-induced acute lung injury

**DOI:** 10.1186/s12950-023-00338-x

**Published:** 2023-04-06

**Authors:** Yeomyeong Kim, Cho-Rong Bae, Dongyeop Kim, Hyejeong Kim, Sunghye Lee, Haiying Zhang, Minyoung Noh, Young-Myeong Kim, Naoki Mochizuki, Young-Guen Kwon

**Affiliations:** 1 Department of Biochemistry, College of Life Science and Biotechnology, Yonsei University, Seoul, 03722 Republic of Korea; 2 Department of Bio Research, Curacle Co. Ltd, Seoul, 06694 Republic of Korea; 3 Department of Molecular and Cellular Biochemistry, School of Medicine, Kangwon National University, Chuncheon, 24341 Republic of Korea; 4 Department of Cell Biology, National Cerebral and Cardiovascular Center Research Institute, 6-1 Kishibe- shimmachi, Suita, Osaka 564-8565 Japan

**Keywords:** Acute lung injury, Lipopolysaccharide, CU06-1004, Inflammation, Endothelial dysfunction

## Abstract

**Background:**

Acute lung injury (ALI) is a life-threatening condition that fundamentally results from inflammation and edema in the lung. There are no effective treatments available for clinical use. Previously, we found that as a leakage blocker CU06-1004 prevents endothelial barrier disruption and enhances endothelial cell survival under inflammatory conditions. In this study, we aimed to elucidate the effect of CU06-1004 in terms of prevention of inflammation and endothelial dysfunction in an ALI mouse model.

**Methods:**

An ALI model was established that included intraperitoneal administration of LPS. Following LPS administration, survival rates and lung wet/dry ratios were assessed. Histological analysis was performed using hematoxylin and eosin staining. Scanning electron microscopy was used to examine alveolar and capillary morphology. Cytokines such as IL-1β, IL-6, and TNF-α were analyzed using an ELISA assay of bronchoalveolar lavage fluid (BALF) and serum. Neutrophil infiltration was observed in BALF using Wright-Giemsa staining, and myeloperoxidase (MPO) activity was assessed. Pulmonary vascular leakage was confirmed using Evans-blue dye, and the expression of junctional proteins was evaluated using immunofluorescent staining. Expression of adhesion molecules was observed using immunofluorescence staining. NF-κB activation was determined using immunohistochemistry and western blot analysis.

**Results:**

Survival rates and pulmonary edema were ameliorated with CU06-1004 treatment. Administration of CU06-1004 normalized histopathological changes induced by LPS, and alveolar-capillary wall thickening was reduced. Compared with the LPS-challenged group, after CU06-1004 treatment, the infiltration of immune cells was decreased in the BALF, and MPO activity in lung tissue was reduced. Similarly, in the CU06-1004 treatment group, pro-inflammatory cytokines were significantly inhibited in both BALF and serum. Evans-blue leakage was reduced, and the expression of junctional proteins was recovered in the CU06-1004 group. Adhesion molecules were downregulated and NF-κB activation was inhibited after CU06-1004 treatment.

**Conclusions:**

These results suggested that CU06-1004 had a therapeutic effect against LPS-induced ALI via alleviation of the inflammatory response and protection of vascular integrity.

**Supplementary Information:**

The online version contains supplementary material available at 10.1186/s12950-023-00338-x.

## Background

Acute lung injury (ALI) is a severe acute disease accompanied by a systemic inflammatory response. Symptoms are associated with lung tissue inflammation and pulmonary edema [1, 2]. The causes of ALI include aspiration of toxic substances, sepsis, trauma, massive blood transfusion, and aspiration pneumonia [3–5]. Commonly, the abnormally increased permeability between alveoli and capillaries results in pulmonary edema [6]. Both the edema and inflammation cause hypoxic respiratory failure [7]. Excessive pro-inflammatory cytokines are expressed, antioxidant enzymes are depleted, and large numbers of neutrophils infiltrate into the alveoli, due to the increased permeability of damaged pulmonary vessels [8, 9]. These changes eventually inhibit gas exchange [9]. About 200,000 cases of ALI occur annually in the USA, with an associated annual mortality rate of 30–40% [1, 10]. Glucocorticoids, inhaled nitric oxide, and activated protein C are used for treatment of ALI, but their clinical efficacy is low [11]. As the COVID-19 pandemic has emerged as a major cause of death for severely ill patients with ALI, effective therapeutic agents are needed [7, 12].

The glycolipid, lipopolysaccharide (LPS), is expressed in the cell walls of Gram-negative bacteria [13]. Because LPS causes inflammation and lung damage, it is widely used for the study of ALI in animal models of sepsis caused by infection [14–16]. LPS forms a complex with LPS binding protein to activate CD14/TLR4 receptors in monocytes and macrophages [17, 18]. It also has an important role in the pathogenesis of ALI via involvement in signal transduction that regulates inflammatory cytokines (e.g., TNF-α, IL-1β, and IL-6) [19–21]. TNF-α participates in initiation and amplification of an inflammatory response [20, 22]. IL-1β contributes to deterioration of the barrier function between lung epithelial cells and pulmonary vascular endothelial cells [23, 24]. IL-6 has a crucial role in the pathogenesis of ALI as it induces formation of a protein-containing hyaline membrane and pulmonary edema [25, 26]. Therefore, inhibition of the LPS-induced production of these inflammatory cytokines is important for effective treatment of ALI.

CU06-1004 is an effective endothelial dysfunction blocker [27–33]. In particular, vascular endothelial cell permeability is reduced by improving survival of vascular endothelial cells and cortical actin ring formation via activation of cAMP/Rac/cortactin signaling [27, 29]. After CU06-1004 administration, the inflammatory responses induced by LPS are alleviated by suppression of expression of adhesion molecules, such as ICAM-1 and VCAM-1, and regulation of NF-κB activation. In LPS-induced ALI, the onset of severe inflammatory disease due to dysfunction of vascular endothelial cells, which generally induces cell migration and infiltration, is the major cause of the pathological changes [34–36]. The purpose of this study was to examine whether CU06-1004 was effective for the anti-inflammation and maintenance of endothelial integrity required for an ALI treatment.

## Results

### CU06-1004 reduced mortality and attenuated pulmonary damages in LPS-induced acute lung injury model

**Fig. 1:**
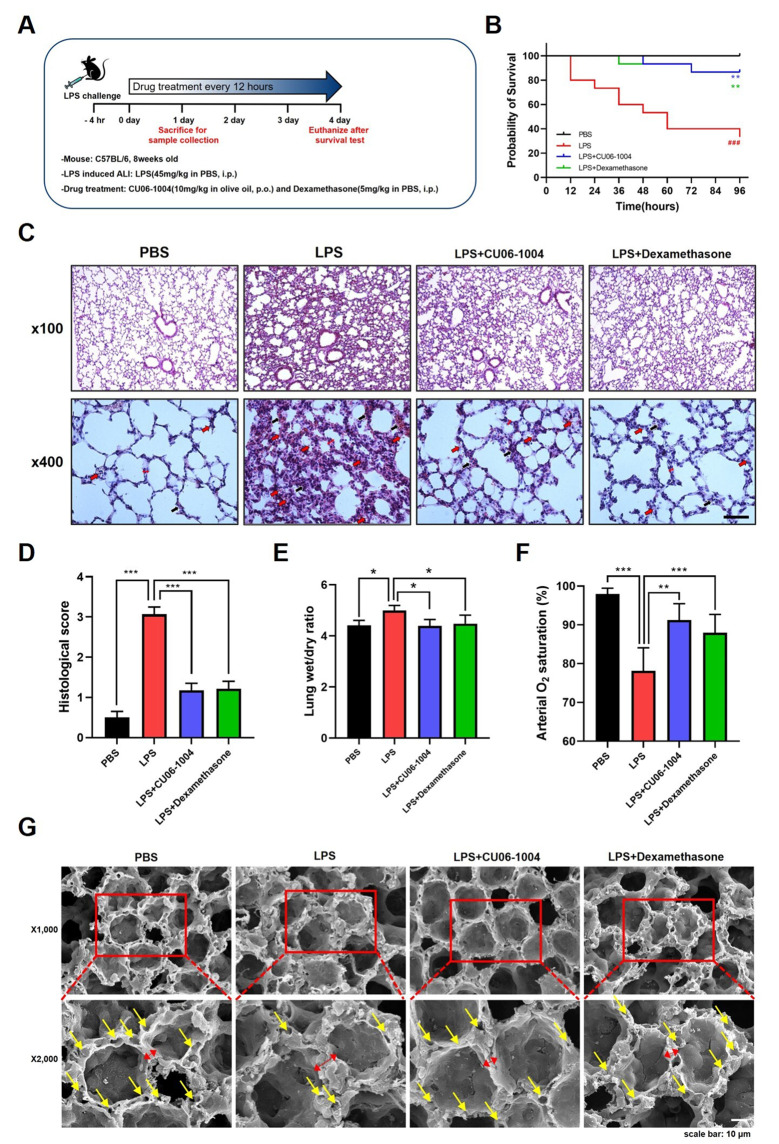
CU06-1004 reduced mortality and attenuated pulmonary damages in LPS-induced acute lung injury model. **(A)** Experimental scheme of study. For assessment of survival, mice were treated with CU06-1004 or dexamethasone every 12 h for 4 days. Sample collections for other tests were performed after one day. The first drug treatment was 4 h after LPS challenge, and mice were sacrificed 4 h after the final treatment. **(B)** The survival rate in each group was determined every 12 h for 4 days after a lethal-dose LPS challenge: PBS group (black line), LPS group (red line), LPS + CU06-1004 group (blue line), and LPS + dexamethasone group (green line). Results expressed as percentage of live mice at each time point. N = 15 per group. ### p < 0.001 vs. PBS, ** p < 0.005 vs. LPS, log-rank tests. **(C)** Representative H&E-stained images of sections from mouse lungs. Lung injury indicated by infiltration of immune cells, interstitial hemorrhage (red arrows), congestion (black arrows), and thickened alveolar walls (two headed arrows, red). N = 6 per group. Magnification, 100X (upper panel) and 400X (lower panel). Scale bar: 30 μm. **(D)** Histological scores evaluated according to the grades as described in methods section. Results presented as mean ± SEM values; *** p < 0.001. **(E)** Lung wet/dry weight ratios determined one day after LPS challenge. N = 8 per group. Results presented as mean ± SD values; * p < 0.05. **(F)** Arterial oxygen concentration was measured by pulse oximeter. N = 5 per group, Results presented as mean ± SD values; ** p < 0.01, *** p < 0.001. **(G)** scanning electron microscopy images, low magnification (1,000X, upper panel) and high magnification (2,000X, lower panel). Normal pulmonary capillaries (yellow arrows) and alveolar wall thickening (two-headed arrow, red) were shown. Scale bar: 10 μm

To determine the therapeutic effect of CU06-1004 in LPS-induced ALI, we investigated whether CU06-1004 improved survival rates in mice with ALI induced by a lethal dose of LPS. The mice were injected with LPS (intraperitoneal route), and mortality was recorded for 4 days. Four hours after the LPS challenge, treatment with CU06-1004 was initiated and given every 12 h at a dose of 10 mg/kg; dexamethasone was given to the positive control mice (Fig. 1A). By 4 days, only 33.3% of the mice survived in the LPS group; in the CU06-1004 (10 mg/kg) and dexamethasone (5 mg/kg) groups, the survival rates were 80% (Fig. 1B). To investigate the effect of CU06-1004 treatment on ALI, pathological changes were examined using hematoxylin and eosin (H&E) staining. After LPS challenge, mice were given CU06-1004 (10 mg/kg) or dexamethasone (5 mg/kg) every 12 h for 1 day and were then euthanized for sample collection (Fig. 1A). Compared with the PBS group, the lung tissues of the LPS group had pulmonary congestion, hemorrhage, and alveolar wall thickening, accompanied by structural damage. However, the LPS-induced pathological changes were attenuated by CU06-1004 or dexamethasone (Fig. 1C). Histological scores were also evaluated to quantify the damage (Fig. 1D). We then determined the microvascular permeability and lung edema caused by LPS, and the wet/dry ratios were calculated. LPS caused an increase in the mean wet/dry ratio, compared with the PBS group. Mean lung wet/dry ratios were significantly decreased both in the LPS + CU06-1004 and LPS + dexamethasone groups, compared with the LPS group (Fig. 1E).In addition, LPS group exhibited markedly reduced arterial oxygen concentration, that was recovered in LPS + CU06-1004 and LPS + dexamethasone groups (Fig. 1F). Scanning electron microscopy revealed that after the LPS challenge the pulmonary capillaries were abnormally shaped and alveolar-capillary walls were thickened. However, CU06-1004 or dexamethasone treatment ameliorated the vascular abnormality and thickened alveolar-capillary wall changes (Fig. 1G). These results indicated that CU06-1004 increased survival rate and reversed the pathological changes that occurred in lung tissue after damage by LPS.

### Effects of CU06-1004 on immune cell infiltration and myeloperoxidase activity

**Fig. 2:**
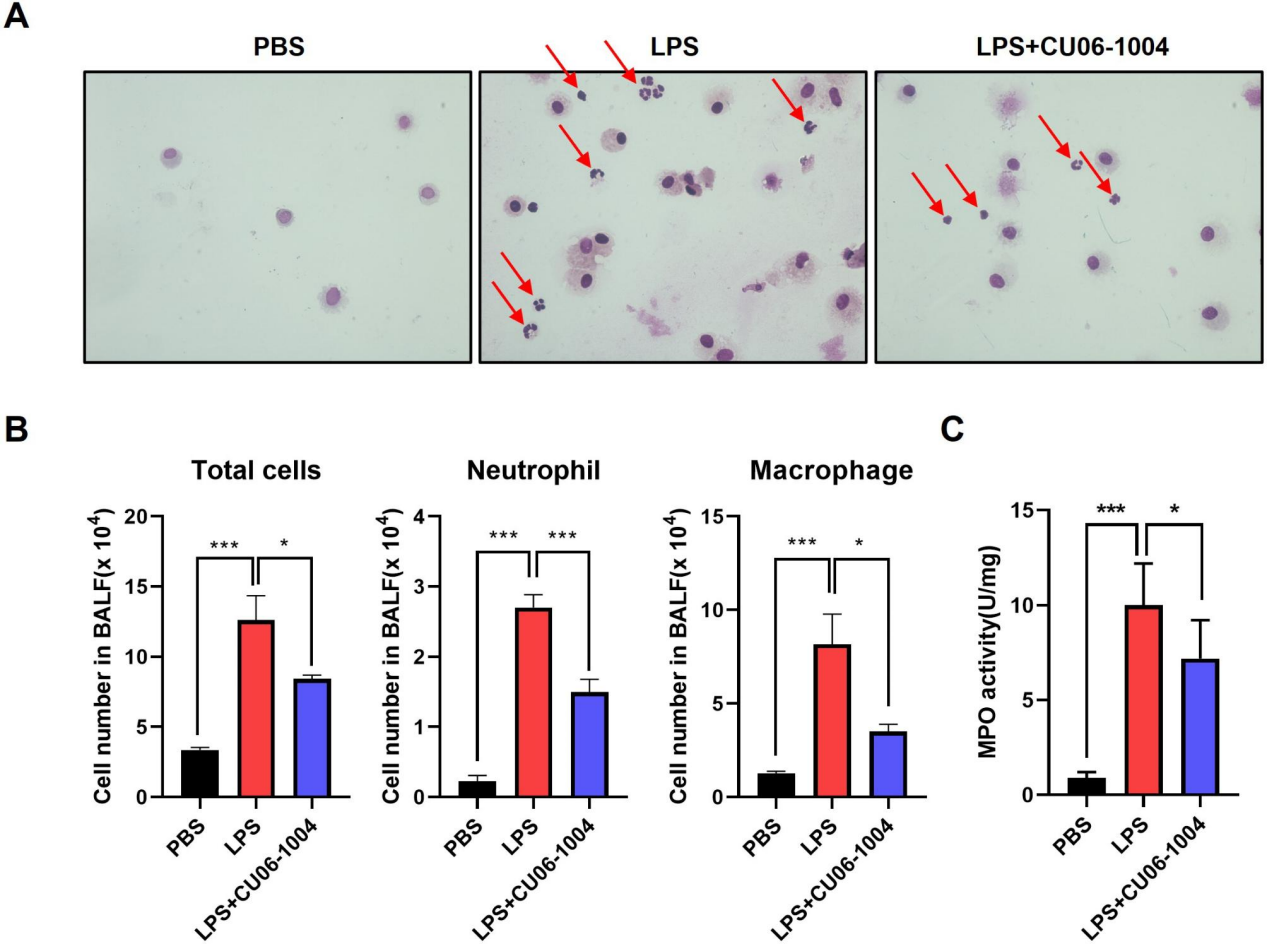
Effects of CU06-1004 on immune cell infiltration and myeloperoxidase (MPO) activity in LPS-challenged mice. **(A)** Representative images of Wright-Giemsa staining using bronchoalveolar lavage fluid (BALF) sample, prepared using cytospin. Neutrophils (red arrows) and macrophages present in BALF. **(B)** Changes in numbers of total cells, neutrophils, and macrophages in BALF. N = 5 per group. **(C)** Effects of CU06-1004 on MPO activity in lung tissue. MPO activity in homogenized lung tissues measured with a microplate reader at 460 nm. N = 6 per group. Results presented as mean ± SEM values; * p < 0.05, *** p < 0.001

To examine the effect of CU06-1004 on changes in inflammatory cells in bronchoalveolar lavage fluid (BALF), samples were attached to a slide using a cytospin and were stained using Wright-Giemsa stain. The BALF samples from the PBS group mice mainly contained alveolar macrophages; neutrophils and macrophages were the predominant types found in the LPS group mice. However, CU06-1004 effectively inhibited the LPS-induced increases in these cells (Fig. 2A). The numbers of total cells, neutrophils, and macrophages in the BALF samples were measured. In the CU06-1004 group, the numbers of total cells, macrophages, and neutrophils were reduced, compared with the LPS group (Fig. 2B). The peroxidase enzyme, myeloperoxidase (MPO), is expressed in neutrophil granulocytes. MPO activity is a biomarker of the progression of LPS-induced ALI. The results indicated that LPS caused a significant mean increase in MPO activity, compared with the PBS control group. CU06-1004 (10 mg/kg) administration inhibited this increase, compared with the LPS group (Fig. 2C). Pulmonary vessels in the lung samples of LPS-induced mice were examined using a scanning electron microscope. In the LPS group, numerous immune cells were attached to the lumen walls of the vessels; these cells were not present after CU06-1004 treatment (Supplementary Fig. 1 in Additional file 1).

### CU06-1004 reduced LPS-induced cytokine levels in bronchioalveolar lavage fluid ,serum and lung lysate

**Fig. 3:**
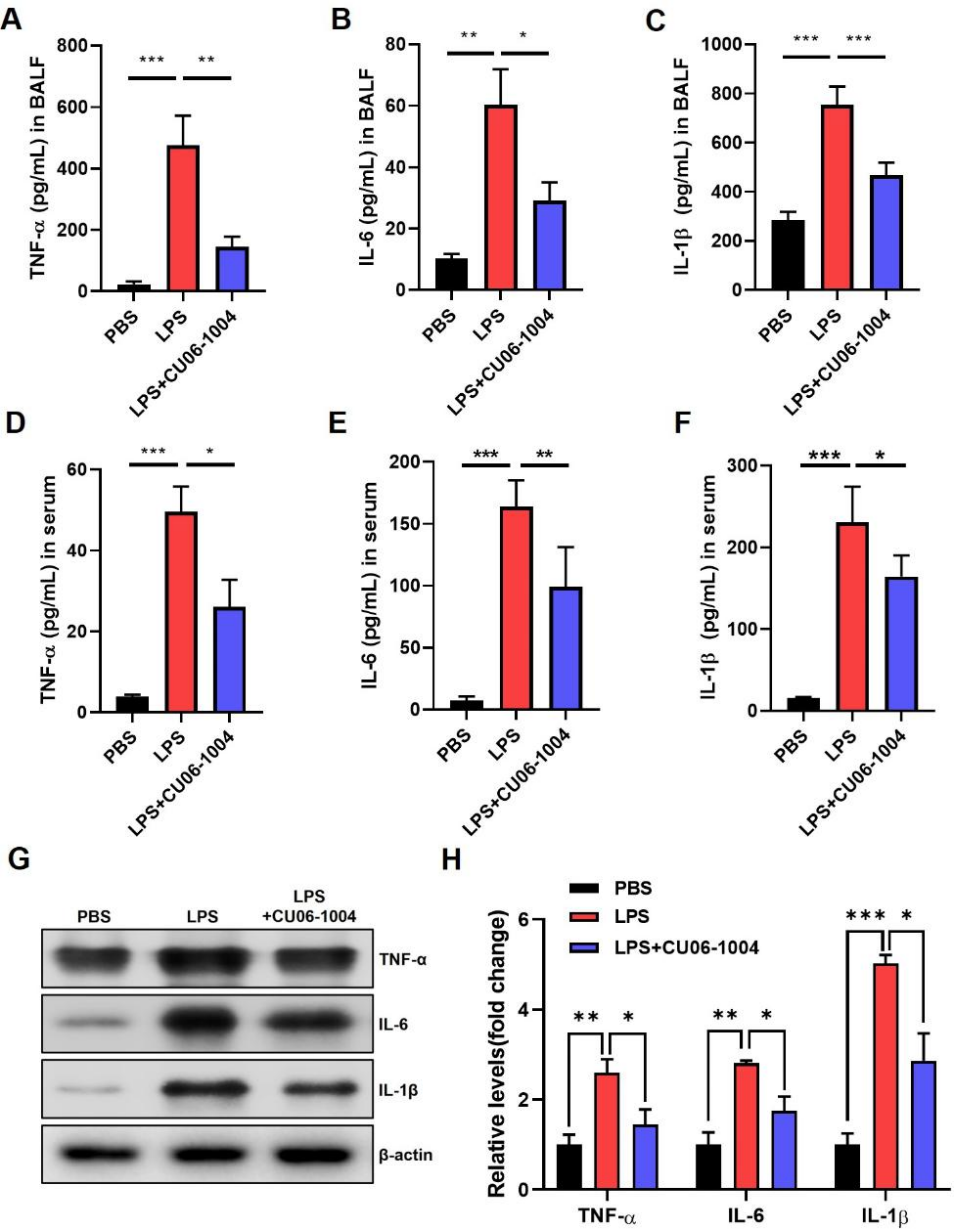
CU06-1004 reduced LPS-induced cytokine levels in bronchoalveolar lavage fluid (BALF), serum, and lung tissue. **(A–C)** BALF collected 32 h after LPS treatment. Inflammatory cytokines in BALF, including (A) TNF-α, (B) IL-6, and (C) IL-1β, measured using ELISA. **(D–F)** Cytokines in serum measured using ELISA, including (D) TNF-α, (E) IL-6, and (F) IL-1β, after whole-blood collection in the left ventricle. N = 4–6 per group. **(G)** Western blot analysis of TNF-α, IL-6, and IL-1β in mouse lung lysate; β-actin used as the loading control. **(H)** Quantitative graph of western blot analysis using ImageJ software as fold change. Results presented as mean ± SEM values; * p < 0.05, ** < p 0.01, *** p < 0.001

To examine the efficacy of CU06-1004 on production of inflammatory cytokines, such as TNF-α, IL-6, and IL-1β, BALF and serum samples were analyzed using ELISA. Cytokine levels were significantly increased in BALF and in serum after LPS challenge, compared with the PBS group. However, mice treated with CU06-1004 had significant decreases in TNF-α, IL-6, and IL-1β in both BALF (Fig. 3A–C) and serum samples (Fig. 3D–F). Moreover, western blot analysis was determined using lung lysate. Pro-inflammatory cytokines were reduced by CU06-1004 treatment after LPS challenge (Fig. 3G, H).

### CU06-1004 ameliorated LPS-induced pulmonary leakage by junctional protein restoration

**Fig. 4:**
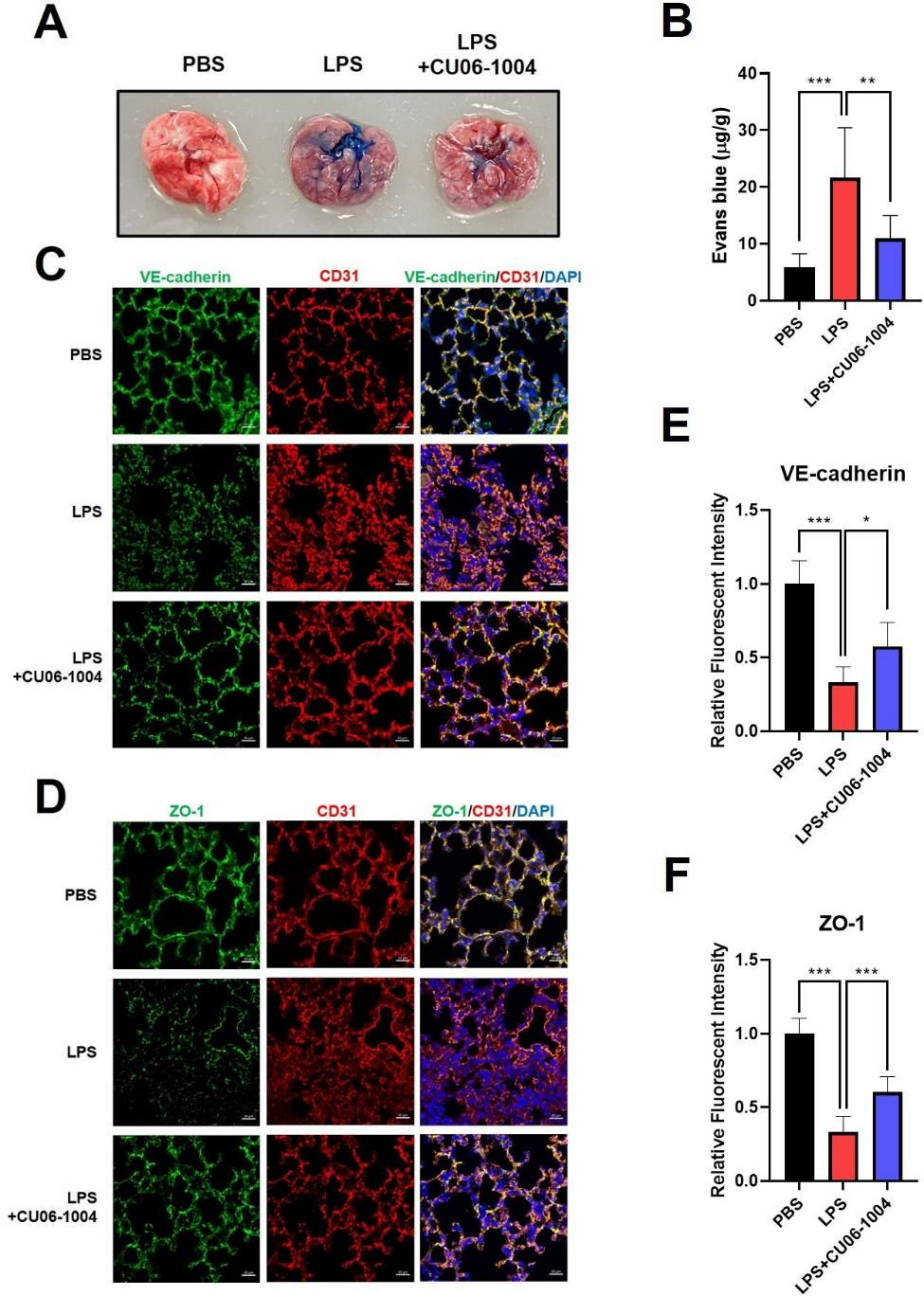
LPS-induced pulmonary leakage was ameliorated by restoring junctional protein after CU06-1004 treatment. **(A)** Representative image of extravasation of Evans-blue dye into the lung and **(B)** quantitative results for Evans-blue dye, measured using spectrophotometry, absorbance at 620 nm. N = 8–10 per group. **(C, D)** Immunofluorescence staining images of intercellular junctions (green) in sections of lung, (C) adherent junction; VE-cadherin and (D) tight junction; ZO-1, with endothelial marker; CD31 (red), and DAPI (blue), confocal microscopy. Scale bar: 20 μm. **(E, F)** Quantitative results for (E) VE-cadherin and (F) ZO-1 expression assessed based on relative fluorescent intensity using ZEN software. N = 6–10 per group. Results presented as mean ± SEM values; * p < 0.05, ** < p 0.01, *** p < 0.001

Extravasation of Evans-blue dye into lung tissue indicates the presence of LPS-induced vascular permeability and immune cell infiltration. We used the Evans-blue leakage assay to evaluate the efficacy of CU06-1004 as a leakage blocker. In the LPS group, the Evans-blue dye was extravasated into lung tissue. However, CU06-1004 reduced the leakage of Evans-blue dye (Fig. 4A, B). Expression of junctional proteins, such as VE-cadherin and ZO-1, was evaluated to determine the anti-permeable effect of CU06-1004 via upregulation of endothelial junctions visible using immunofluorescent staining (Fig. 4C, D). In the LPS group, protein expression of VE-cadherin and ZO-1 was diminished, compared with the PBS group. However, the quantitative analysis revealed the LPS + CU06-1004 group had significantly increased expression of junctional proteins (Fig. 4E, F). The transmission electron microscopy morphology results indicated that after LPS challenge the endothelial cell-to-cell contacts that formed the pulmonary vascular lumen were weakened; the endothelial cells were also swollen. CU06-1004 treatment restored these changes in cell morphology (Supplementary Fig. 2 in Additional file 1).

### CU06-1004 downregulated expression of adhesion molecules in lungs with LPS-induced acute lung injury

**Fig. 5:**
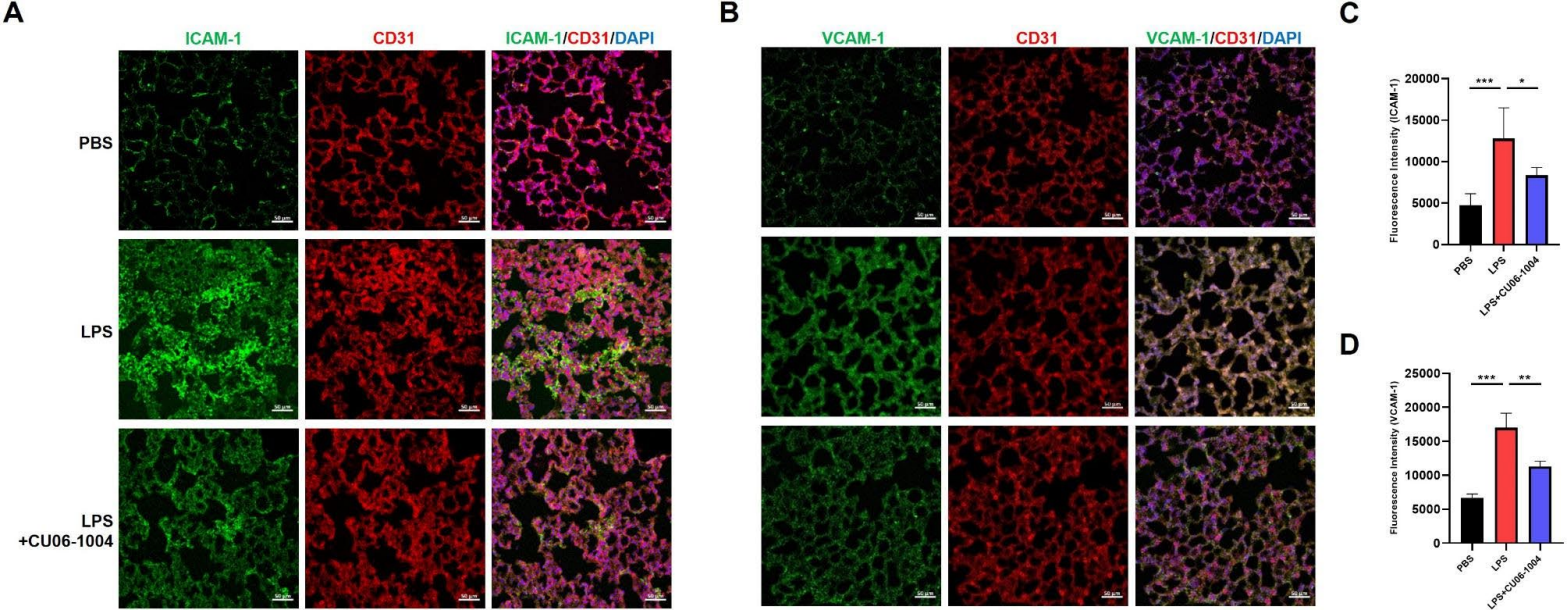
CU06-1004 downregulated expression of adhesion molecules in LPS-induced ALI. **(A, B)** Immunofluorescence staining for (A) ICAM-1 and (B) VCAM-1 (green) with CD31 (red), and DAPI (blue) in cryo-section of lung at 32 h after LPS challenge. Scale bar: 50 μm. **(C, D)** Quantitative analysis of (C) ICAM-1 and (D) VCAM-1 expression based on calculation of intensity of fluorescence using ZEN software. N = 4–5 per group. Results presented as mean ± SEM values; * p < 0.05, ** < p 0.01, *** p < 0.001

Adhesion molecules, such as ICAM-1 and VCAM-1, have a critical role in pulmonary inflammation caused by LPS stimulation [37, 38]. To investigate whether there was an increase in adhesion molecule expression, immunofluorescent staining was evaluated using fluorescence intensity. In the LPS group, expression of ICAM-1 and VCAM-1 in the lung was markedly increased, compared with the PBS group. However, CU06-1004 significantly downregulated expression of adhesion molecules, compared with the LPS group (Fig. 5A, B). These results were presented as quantitative graphs of changes in fluorescent intensity (Fig. 5C, D).

### CU06-1004 reduced NF-ĸB p65 activation in LPS-induced lung tissue injury

**Fig. 6:**
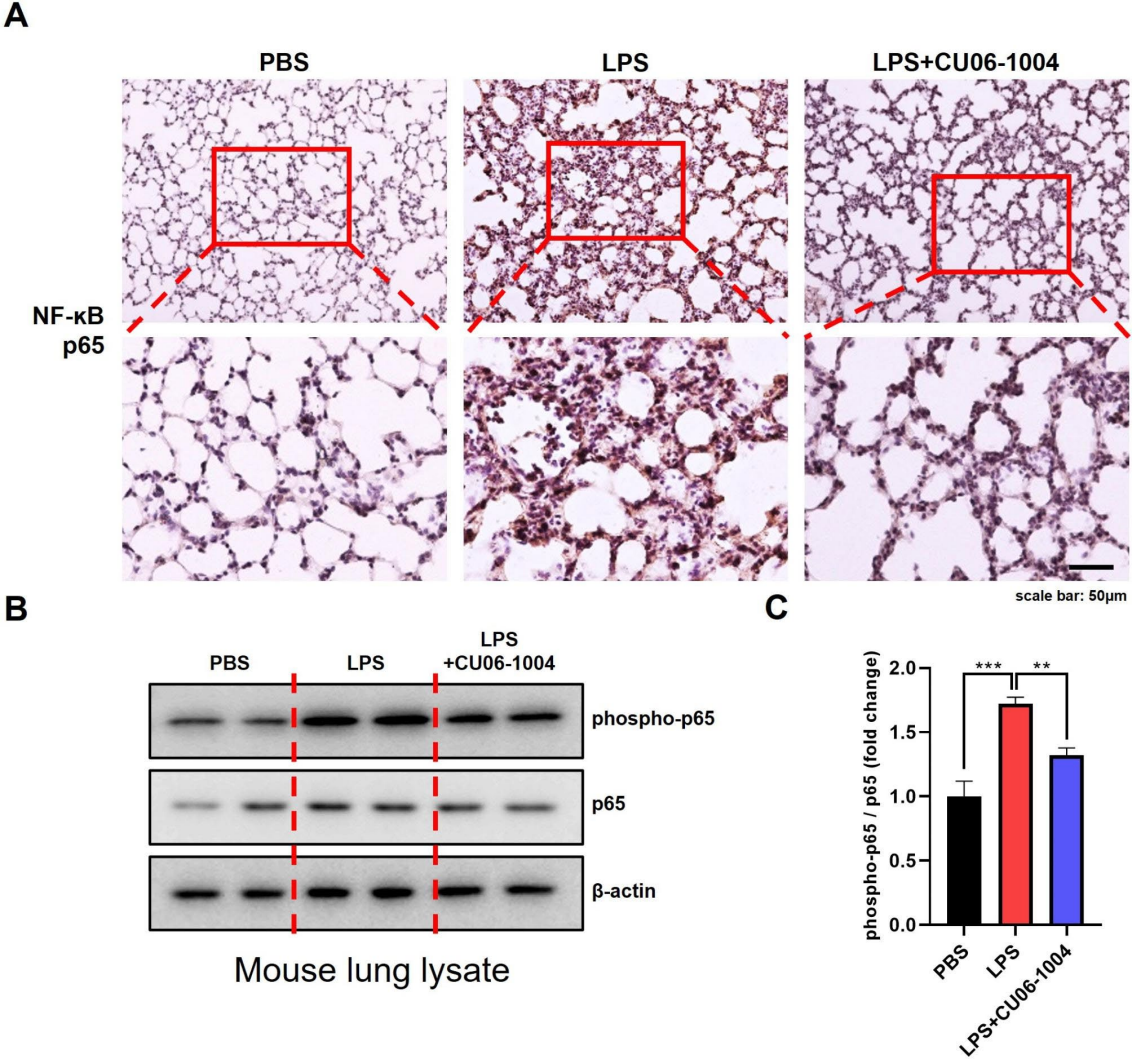
CU06-1004 reduced NF-ĸB p65 activation in LPS-induced ALI. **(A)** Lung tissues from LPS-challenged mice showed NF-κB p65-positive staining using immunohistochemistry. Magnification, 200X (upper panel) and 400X (lower panel). Scale bar: 50 μm. **(B)** Expression of phosphor-NF-κB p65 and NF-κB p65 in mouse lung lysate was analyzed using western blotting; β-actin used as the loading control. **(C)** Quantitative graph of western blot analysis using ImageJ software as phospho-p65 per p65. N = 6 per group. Results presented as mean ± SEM values; ** p < 0.01, *** p < 0.001

To evaluate expression of NF-ĸB p65, an IHC assay was used to examine lung tissue sections from the LPS-induced mice with ALI. Lung tissue samples from the LPS group had significantly positive staining of NF-ĸB p65, compared with the PBS group mice. In contrast, lung tissue from mice challenged with LPS and then treated with CU06-1004 had reduced expression of NF-ĸB p65 (Fig. 6A). The lysates of lung tissue from LPS-induced mice were used for western blot analysis to confirm activation of NF-ĸB p65. Protein expression of the phosphorylated and total forms of NF-ĸB p65 was evaluated using β-actin (Fig. 6B). Expression of both forms of NF-ĸB p65 was elevated in the LPS group, compared with the PBS group mice. However, CU06-1004 decreased expression of phosphorylated and total NF-ĸB p65. The results for the ratios of phosphorylated NF-ĸB p65 to total NF-ĸB p65 are presented in Fig. 6C.

## Discussion

ALI involves inflammation-induced endothelial dysfunction, alveolar-capillary barrier integrity, and pulmonary edema that can lead to the clinical symptoms and signs of poor lung compliance, severe hypoxemia, and bilateral infiltrates on chest radiographs [1, 39, 40]. It is caused by immune cell infiltration of alveoli; these immune cells release inflammatory cytokines [41]. These released cytokines damage pulmonary endothelial cells and disrupt pulmonary epithelial integrity [42, 43]. Finally, pulmonary vascular permeability is increased, and alveolar gas exchange is impaired [44].

Diverse preclinical disease models reveal that CU06-1004 is a potent and efficient endothelial dysfunction blocker. CU06-1004 activates Rac, a small GTPase, and stabilizes junctional molecules and the structure of cortical actin rings via the cAMP/Rac/cortactin pathway present in endothelial cells [27]. A middle cerebral artery occlusion murine model found that as a vascular leakage blocker CU06-1004 protects cerebral ischemia-reperfusion injury by suppressing endothelial junctional breakage and inflammation [29, 45]. It also ameliorates dextran sodium sulfate-induced colitis and thus inhibits loss of gut epithelial barrier integrity via stabilization of the endothelial junction and inflammatory response [30]. Combination therapy using CU06-1004 and anti-PD-1 antibody results in significantly improved immunotherapy via normalization of tumor vessels [31].

The endotoxin LPS is present in the cell walls of Gram-negative bacteria and is typically used to establish in-vivo ALI models [13, 16]. LPS disrupts endothelial barrier function and leads to increases in inflammatory cytokines, such as IL-1 and TNF-a, which have an important role in the pathogenesis of ALI [46, 47]. In this study using an LPS-induced ALI mouse model, we found that CU06-1004 had a therapeutic effect by blocking endothelial dysfunction and inhibiting inflammation.

Although numerous emerging treatments have been studied, the mortality rate of ALI remains up to 30–40% [48]. Therefore, we also used the model to evaluate survival rate. Survival rate was about 33.3% by 4 days in the LPS group when a lethal dose (45 mg/kg) was given. However, post-treatment with CU06-1004 or dexamethasone significantly reduced mortality rate (about 86.7% survival in the CU06-1004 10 mg/kg and dexamethasone 5 mg/kg groups). This result indicated that CU06-1004 had a therapeutic effect that improved the survival rate associated with LPS-induced ALI.

Pulmonary edema is a well-known clinical sign of ALI and causes respiratory failure, which is the main contributor to ALI-associated death [49–51]. To evaluate lung edema, we measured wet/dry ratios in lung tissue. In the LPS + CU06-1004 group, the lung wet/dry ratio was decreased. After euthanized, the visual appearance of lung tissue showed a lot of hemorrhagic regions in LPS group, and CU06-1004 decreased hemorrhage. However, the edema of other tissues, such as kidney, spleen, and liver was not shown with acute LPS challenge (Supplementary Fig. 3A, B in additional file 1). This result suggested that CU06-1004 protected the lung from acute pulmonary microvascular leakage. We also evaluated histopathological changes. The LPS group had infiltration of immune cells, hemorrhage, congestion of alveolar capillaries, and alveolar wall thickening. In contrast, the LPS + CU06-1004 and LPS + dexamethasone groups had attenuated histological damage. These results suggested that CU06-1004 had a positive effect on LPS-induced ALI. To further examine the structure of pulmonary alveoli and the endothelium, lung tissues from LPS-challenged mice were evaluated using transmission electron microscopy and scanning electron microscopy. In the LPS group, the endothelial junctions of the pulmonary capillaries were attenuated, and the endothelial cells were swollen. However, the LPS + CU06-1004 group had strengthened endothelial junctions and normalization of cell morphology (Supplementary Fig. 2 in additional file 1). The scanning electron microscopy results revealed that in the LPS group the pulmonary alveolar wall thickness was increased and tortuous microvessels were present. Many immune cells were also attached to the pulmonary vascular wall after LPS challenge. However, alveolar morphology and the attached immune cells were normalized with CU06-1004 treatment (Supplementary Fig. 1 in additional file 1). LPS induces tissue injury and endothelial barrier dysfunction, which leads to vascular leakage [46]. Enhancement of cellular connections affected by cell-cell adhesion is important for inhibition of endothelial barrier disruption [46, 52]. In the endothelium, the major intercellular proteins include VE-cadherin, ZO-1, claudin-5, and connexin-43; these proteins regulate vascular permeability [53]. VE-cadherin is a main transmembrane protein of endothelial cells; it has a key role in maintenance of endothelial permeability [54]. ZO-1 controls the paracellular permeability of membranes to prevent attacks by infectious agents [55]. To examine whether CU06-1004 protected junctional integrity, Evans-blue dye was injected into LPS-treated mice and extravasation of dye was measured in lung tissues. Compared with the LPS group, the LPS + CU06-1004 group had reduced leakage of Evans-blue dye. This result indicated that CU06-1004 inhibited vascular permeability. Expression of junctional proteins was evaluated using immunofluorescence staining, and CU06-1004 upregulated the expression of VE-cadherin and ZO-1 that were reduced by LPS treatment. Therefore, we found that CU06-1004 protected pulmonary vascular intensity via regulation of expression of junctional proteins.

MPO activity represents infiltration of neutrophils into the pulmonary tissue, which was used to evaluate tissue damage [56]. CU06-1004 significantly reduced MPO activity in the lung tissue. This result indicated that CU06-1004 inhibited neutrophil infiltration. This result was consistent with the results of the Wright-Giemsa staining used to evaluate infiltration of immune cells in BALF. These results may be due to an anti-inflammatory effect of CU06-1004 on LPS-induced mouse lung tissue. LPS markedly increased expression of inflammatory cytokines, such as TNF-α, IL-1β, and IL-6, that are involved in the pathological progression of ALI [25]. TNF-α is an important mediator of inflammation and is initially produced in LPS-induced ALI [57]. IL-1β, another pro-inflammatory cytokine, causes multiple organ failure associated with endotoxic shock [24]. IL-6 also has a crucial role in LPS-induced inflammation and is used as a marker in murine ALI models [49]. In this study, we evaluated the therapeutic effects of CU06-1004 in LPS-induced ALI, in BALF, serum, and lung tissue. The results indicated that CU06-1004 significantly reduced pro-inflammatory cytokines during progression of ALI. The cell adhesion molecules, ICAM-1 and VCAM-1, have a pivotal role in pulmonary inflammation by promoting the migration (and subsequent adhesion) of leukocytes into the lung [57, 58]. Immunofluorescence staining revealed that LPS elevated expression of ICAM-1 and VCAM-1. However, CU06-1004 ameliorated the expression of adhesion molecules. NF-κB is a major transcription factor and has a regulatory role during production of inflammatory cytokines [59]. NF-κB regulates transcription of adhesion molecules and promotes expression of ICAM-1 and VCAM-1 in endothelial cells [60]. LPS stimulates NF-κB activation, which then translocates to the nucleus and induces an inflammatory response [61, 62]. In our previous study, we found that CU06-1004 has an anti-inflammatory mechanism by inhibiting NF-κB activation in endothelial cells stimulated with IL-1β [29]. Here, we confirmed that CU06-1004 significantly reduced NF-κB activation in mouse model of LPS-induced ALI. Further studies require more in-depth investigation to elucidate the molecular mechanism of CU06-1004. However, we found that CU06-1004 had anti-inflammatory effects on LPS-induced ALI.

## Conclusions

This study revealed that CU06-1004 had anti-inflammatory effects on LPS-induced ALI by suppressing activation of NF-ĸB and downregulating expression of adhesion molecules. CU06-1004 also protected endothelial junctional integrity that was damaged by LPS-induced lung injury. These results suggested that CU06-1004 is a potential therapeutic agent for treatment of ALI.

## Methods

### Chemical and drugs

CU06-1004 was synthesized as previously described [27]. Briefly, CU06-1004 was synthesized via tetrahydropyran deprotection and subsequent glycosidation with 4,6-di-O-acetyl-2,3-didieoxyhex-2-enopyran in the presence of acid. LPS and dexamethasone were purchased from Sigma Aldrich (St. Louis, MO, USA).

### Animals and ethical statement

Eight-week-old male mice (C57BL/6J, 21–23 g body weight) were purchased from DBL (Eumseong, Chungcheongbuk-do, Republic of Korea) and were used for the entire study. All mice were housed in standard cages at constant temperature (22 ± 1 °C) and humidity (55 ± 5%), with a 12-hour light/dark cycle and free access to food and water. All experiments involving animals were approved in advance by the Animal Care and Use Committee of Yonsei University (Seoul, Republic of Korea) and were performed in accordance with approved guidelines (IACUC-A-202010-1151-01).

### LPS challenge and drugs treatment

All mice were acclimated for 1 week and randomly divided four groups: PBS group, LPS group, LPS + CU06-1004 group, and LPS + dexamethasone group. LPS (Sigma Aldrich, St. Louis, USA, L2880) was dissolved in PBS (45 mg/kg) and was injected via the intraperitoneal route. CU06-1004 was dissolved in olive oil (O1514, Sigma Aldrich, St. Louis, USA) and was injected via the oral route (10 mg/kg) 4 h after the LPS challenge. Dexamethasone (Sigma Aldrich, St. Louis, USA, D2915) was dissolved in PBS and injected via the intraperitoneal route (5 mg/kg). The mice in the PBS groups received PBS only, instead of LPS, and an equal volume of olive oil (per os) or PBS (intraperitoneal injection), instead of CU06-1004 or dexamethasone, was given.

### Survival test

To assess survival rates (percent survival) during the entire study period, 75 mice were randomly assigned to one of four groups: PBS, LPS, LPS + CU06-1004 (10 mg/kg), or LPS + dexamethasone (5 mg/kg). Mortality of the mice was recorded every 12 h for 4 days.

### Pulse oximetry

To assess arterial oxygen content, mice were monitored for the percentage of hemoglobin saturated with oxygen. Mice were anesthetized using avertin (2,2,2-tribromoethanol, Sigma Aldrich, USA, T48402) at 240 mg/kg, and the fur on the neck was removed by shaving. The sensor was placed on the skin, and three consecutive sustained readings were averaged using MouseOx Plus oximeter (Starr Life Sciences, Oakmont, PA, USA).

### Tissue wet/dry ratio

All mice were euthanized a day after the LPS challenge. The tissue wet weights were recorded after tissues were excised. The tissues were then placed in an oven at 60 ℃ for 48 h, and then the dried tissue weights were recorded. Wet/dry ratios were calculated by dividing dry weight by wet weight.

### Tissue collection

At the end of the experiment, all mice were anesthetized using avertin (2,2,2-tribromoethanol, Sigma Aldrich, USA, T48402) at 240 mg/kg, and a CO2 chamber was used to euthanize the animals. The lungs were dissected, transferred to 4% PFA for 1–2 days, and then used for the subsequent experiments.

### Histopathological analysis

Fixed lung tissues were paraffinized using a tissue processor (Leica, Wetzlar, Germany, 0422). The tissues then were sectioned at 5 μm using a microtome (Leica, Wetzlar, Germany, 9224) and were stained using an H&E staining kit (Abcam, Dawinbio Inc., Hanam, Gyeonggi-do, Republic of Korea, ab245880). The stained samples were examined under an optical microscope (Nikon, Tokyo, Japan, Eclipse 80i), and the images were graded from 0 to 4. The grades used were: 0, no injury (normal); 1, minimal (injury up to 25% of the field); 2, mild (injury between 25 and 50% of the field); 3, moderate (injury between 50 and 75% of the field); and 4, severe (injury over 75% of the field), for thickening of the alveolar-capillary walls, the numbers of infiltrated cells, hemorrhage, and the vascular congestion.

### Immunohistochemistry analysis

Each tissue section was de-paraffinized using xylene (three times) and serial alcohol (100%, 90%, 80%, 70%, and 50%) and then exposed to citrate butter (10mM sodium citrate acid, 0.05% Tween20, pH 6.0) for antigen retrieval. The section was blocked with blocking solution (DAKO, Santa Clara, CA, USA, X0909) for 1 hour at room temperature and was incubated overnight at 4°C with primary antibodies. The primary antibody was NF-κB p65 antibody (Thermo Fisher Scientific, IL, USA, 33-9900). An EnVision Detection Kit (DAKO, Santa Clara, CA, USA, K5007) was used for secondary-peroxidase complex and 3,3’-diaminobenzidine tetrahydrochloride salt staining. Stained samples were examined under an optical microscope (Nikon, Tokyo, Japan, Eclipse 80i).

### Scanning electron microscopy

Lung tissue from mice was cut into longitudinally through the lumen of the mediastinal airways extending from the lobar bronchus to the distal airways. Specimens were fixed for 24 h in Karnovsky’s fixative (2% Glutaraldehyde, 2% Paraformaldehyde in 0.1 M phosphate buffer, pH 7.4) and washing two times for 30 min in 0.1 M PBS. They were postfixed with 1% osmium tetroxide for 2 h and dehydrated in ascending gradual series (50, 60, 70, 80, 90, 100, and 100%) of ethanol and used a Critical Point Dryer (EM CPD300, LEICA, Germany). They were coated with platinum by ion sputter (EM ACE600, LEICA, Germany) and observed with a field emission Scanning electron microscopy (MERLIN, ZEISS, Germany).

### Transmission electron microscopy

For transmission electron microscopy (TEM) lung tissue was obtained from ALI mice and cut into small pieces (1 mm^3^). Specimens were fixed for 12 h in 2% glutaraldehyde at 4 °C, rinsed in 0.1 M phosphate buffer (pH 7.4), followed by post fixation in 1% osmium tetroxide for 2 h, and dehydrated with an ascending ethanol series (50, 60, 70, 80, 90, 95, 100, and 100%) for 10 min each. Specimens were embedded with a Poly/Bed 812 kit (08792-1, Polysciences, PA, USA), polymerized in an electron microscope oven (TD-700, DOSAKA, Japan) at 65℃ for 12 h. The block is equipped with a diamond knife in the ultramicrotome and is cut into 200 nm semi–thin section and stained toluidine blue for observation of optical microscope. The region of interest was then cut into 80 nm thin sections using the ultramicrotome, placed on copper grids, double stained with 3% uranyl acetate for 30 min and 3% Lead citrate for 7 min staining, and imaged with a transmission electron microscopy (JEM-1011, JEOL, Tokyo, Japan) at the acceleration voltage of 80 kV equipped with a Megaview III CCD camera (Soft imaging system-Germany).

### Myeloperoxidase activity

MPO activity was evaluated to assess neutrophilic infiltration to the lung tissue [ ]. The MPO activity was measured using an MPO activity assay kit (Abcam, Dawinbio Inc., Hanam, Gyeonggi-do, Republic of Korea, ab105136), according to the manufacturer’s instructions.

### Collection of bronchoalveolar lavage fluid and serum

BALF and serum samples were collected 32 h after the LPS challenge. Briefly, each mouse was anesthetized using avertin (240 mg/kg) and the middle of the trachea was carefully punctured using a 26-gauge needle. A catheter was then inserted into the trachea and the lungs were washed three times with PBS (0.7 ml each time). For serum collection, whole blood was collected from the heart using a 1 ml syringe. The collected blood was allowed to clot at room temperature for 30 min. The clot was then separated from the supernatant using centrifugation at 1000 × g for 10 min at 4 ℃, and the supernatant was collected.

### Wright-Giemsa staining

Each BALF sample was centrifuged at 300 × g for 10 min in a cytocentrifuge with a slide for attaching the cells. Then, the slide was stained using a Wright-Giemsa Stain Kit (Abcam, Dawinbio Inc., Hanam, Gyeonggi-do, Republic of Korea, ab245888), according to the manufacturer’s instructions.

### Measurement of cytokine levels in bronchoalveolar lavage fluid and serum

The levels of inflammatory cytokines were measured in the BALF and serum samples. TNF-α, IL-1β, and IL-6 were measured using ELISA kits (R&D Systems, Minneapolis, MN, USA, #MTA00B, #M6000B, and #MLB00C), following the manufacturer’s instructions.

### Western blot analysis

Proteins from lung tissues were extracted in radioimmunoprecipitation assay buffer (100 mM Tris-Cl, 5 mM EDTA, 50 mM NaCl, 50 mM β-glycerophosphate, 50 mM NaF, 0.1 mM Na3VO4, 0.5% NP-40, 1% Triton X-100, and 0.5% sodium deoxycholate). Sample protein concentrations were quantified using a SMART BCA Protein Assay kit (iNtRON Biotechnology, Inc., Gyeonggi-do, Korea). Next, tissue lysates were separated using sodium dodecyl sulfate-polyacrylamide gel electrophoresis and then transferred to nitrocellulose membranes. The membranes were blocked with 3% bovine serum albumin in 0.1% tris-buffered saline with Tween 20 (TBST) and then probed with primary antibodies. The membranes were then incubated with horseradish peroxidase-conjugated goat anti-rabbit IgG or goat anti-mouse IgG (Thermo Fisher Scientific, IL, USA, #31,460, #31,430) secondary antibodies. β-Actin was used as a loading control. The primary antibodies used (1:1000 dilution) were: TNF-α (Cell signaling Technology, Danvers, USA, #3707), IL-6 (Cell signaling Technology, Danvers, USA, #12,912), IL-1β (Abcam, Dawinbio Inc., Hanam, Gyeonggi-do, Republic of Korea, ab234437), NF-κB p65 (Cell Signaling Technology, Danvers, USA, #8242), Phospho-NF-κB p65 (Cell Signaling Technology; Danvers, USA, #3031), and β-actin (Thermo Fisher Scientific, IL, USA, MA5-15739).

### Vascular permeability assay

Evans-blue (1% in PBS; Sigma Aldrich, St. Louis, USA, E2129) dye was used to evaluate the pulmonary leakage. Evans-blue dye was injected via the intravenous route 30 min before the lung tissue was collected. Evans-blue leakage was quantified in the lung tissue after the lung was homogenized and incubated in formamide (24 h, 55 °C). The Evans-blue assay result was measured in the supernatant from each sample (absorbance, 620 nm). Results were calculated using a standard curve for Evans-blue in formamide and were presented as micrograms per gram of lung tissue.

### Cryo-section and immunofluorescence staining

Lung tissue was exposed to paraformaldehyde (4% in PBS) for 1 or 2 days (4 °C) for fixation. The tissue was then rinsed with PBS at room temperature, incubated overnight (4 °C) in sucrose (15%), and then transferred to sucrose (30%) at 4 °C until the tissue sank. Tissue-Tek optimum cutting temperature (OCT) embedding medium was then used to infiltrate the fixed lung for 30 min at room temperature. The samples were stored at -80 °C after transfer to an OCT-filled embedding mold and freezing with dry ice. While frozen, Sect. (10 μm-thick) were cut onto slides at -20 °C for immunostaining. The slides were stored at -80 °C until use for this procedure. Briefly, the sections were prefixed in acetone for 30 min at -80 °C and air dried. Flowing water was used to rinse the OCT. The sections were then incubated overnight in primary antibody (1:200, 4 °C), washed three times (5 min per wash) with Triton X-100 (0.1%) in PBS, and were incubated for 2 h in secondary antibody (1:300, room temperature). The sections were then counterstained using DAPI (4′,6-diamidino-2-phenylindole, 1 µg/ml) and were washed three times with Triton X-100 (0.1%) in PBS (5 min per wash). Antibody diluent (Dako, Agilent Technologies, Santa Clara, CA) was used to dissolve each antibody. A confocal microscope (LSM 880, Carl Zeiss) was used to examine each section.

### Statistical analysis

GraphPad Prism 8.0 (GraphPad Software, Inc., La Jolla, CA, USA) was used for the data analysis. The results were presented as mean ± standard deviation or standard error of the mean values. The data were analyzed using one-way analysis of variance, followed by Tukey’s multiple comparison tests. Kaplan-Meier survival curves were compared using log-rank tests. A P < 0.05 was considered to indicate a statistically significant difference.

## Electronic supplementary material

Below is the link to the electronic supplementary material.

**Additional file 1: Supplementary Figure 1.** CU06-1004 reduced attachment of immune cells on pulmonary vascular lumen. **Supplementary Figure 2.** CU06-1004 restored endothelial cell-to-cell contacts and swollen morphology after LPS challenge. **Supplementary Figure 3.** Visual appearance and edema of tissues in mouse.

**Additional file 2:** Original blot images of Figure 6B

## Data Availability

The datasets used and/or analyzed during the current study are available from the corresponding author on reasonable request.

## List of abbreviations

**ALI**: Acute lung injury
**BALF**: Bronchoalveolar lavage fluid
**CD31**: Cluster of differentiation 31
**DAPI**: 4′,6-diamidino-2-phenylindole
**ELISA**: Enzyme-linked immunosorbent assay
**ICAM-1**: Intercellular adhesion molecule-1
**IL-1β**: Interleukin-1 beta
**IL-6**: Interleukin-6
**LPS**: Lipopolysaccharide
**MPO**: Myeloperoxidase
**NF-κB**: Nuclear factor-kappa B
**SEM**: Scanning electron microscopy
**TEM**: Transmission electron microscopy
**TLR4**: Toll-like receptor 4
**TNF-α**: Tumor necrosis factorα
**VCAM-1**: Vascular cell adhesion molecule-1
**VE-cadherin**: Vascular endothelial-cadherin
**ZO-1**: Zonular occludens-1
