# Supplementary material for: Efficacy of CU06-1004 via regulation of inflammation and endothelial permeability in LPS-induced acute lung injury
Source: J Inflamm (Lond). 2023 Apr 6;20:13. doi: 10.1186/s12950-023-00338-x (PMC10078077; doi:10.1186/s12950-023-00338-x)
Supplement: Supplementary file 1 — Additional file 1: Supplementary Figure 1. CU06-1004 reduced attachment of immune cells on pulmonary vascular lumen. Supplementary Figure 2. CU06-1004 restored endothelial cell-to-cell contacts and swollen morphology after LPS challenge. Supplementary Figure 3. Visual appearance and edema of tissues in mouse. [file 12950_2023_338_MOESM1_ESM.docx]

**Supplementary Figure 1**


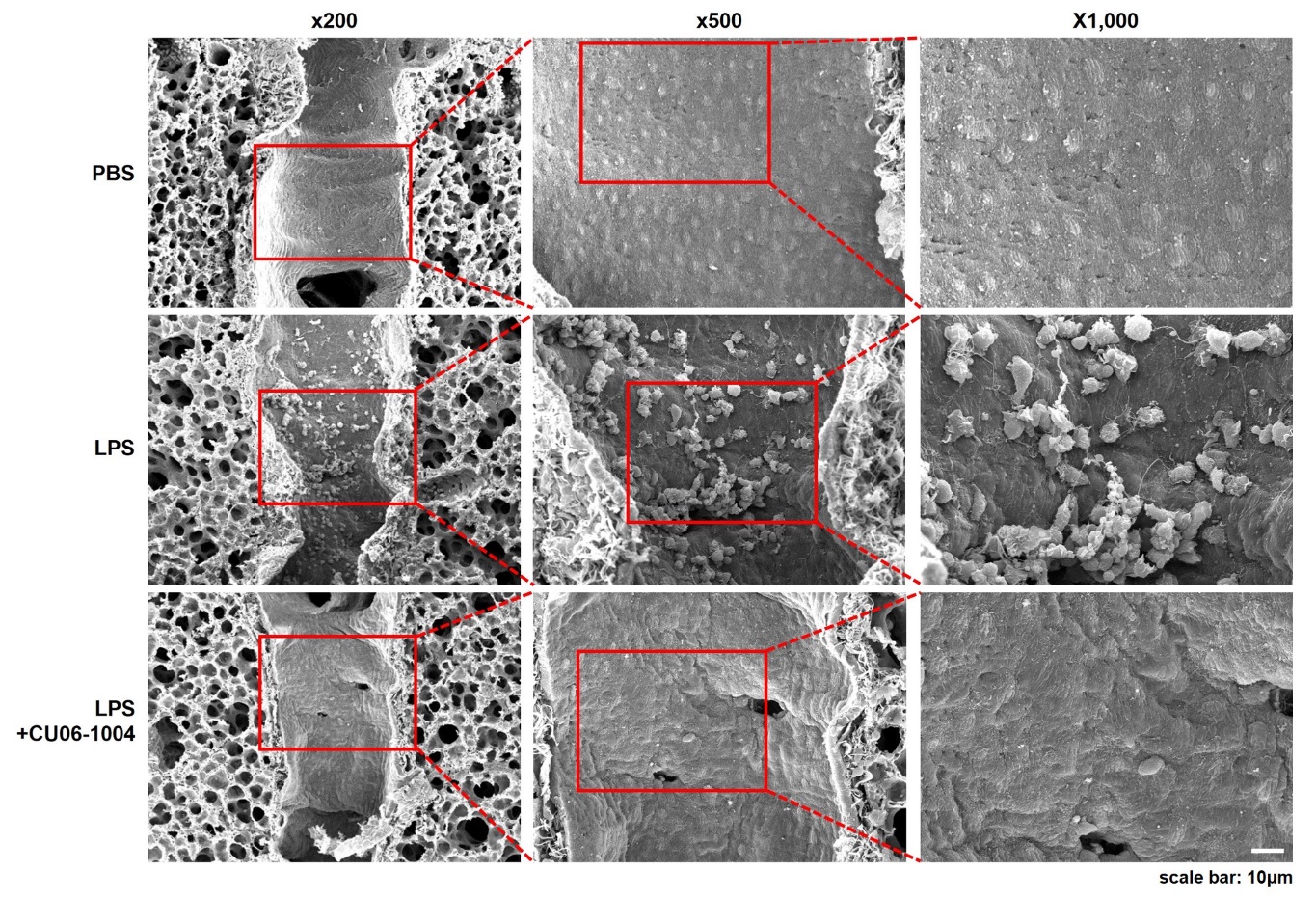


**Supplementary Figure 2**

**
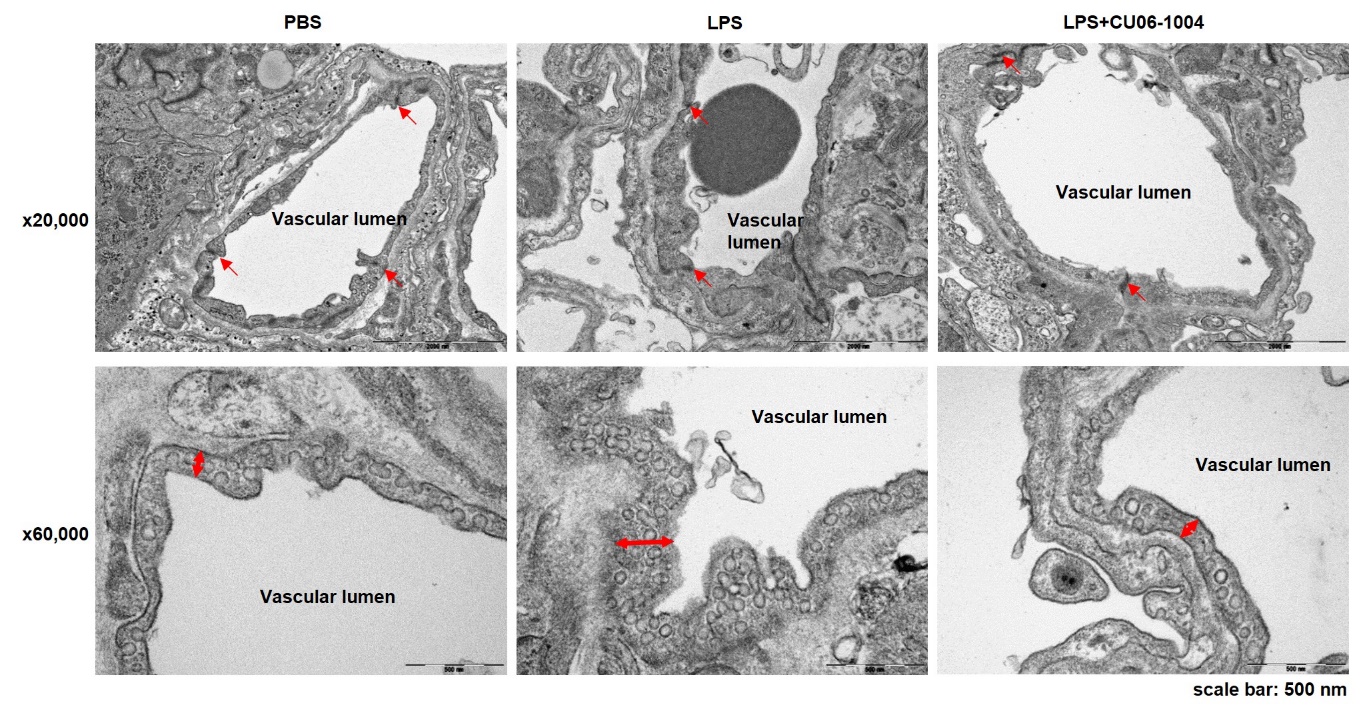
**

**Supplementary Figure 3**

**
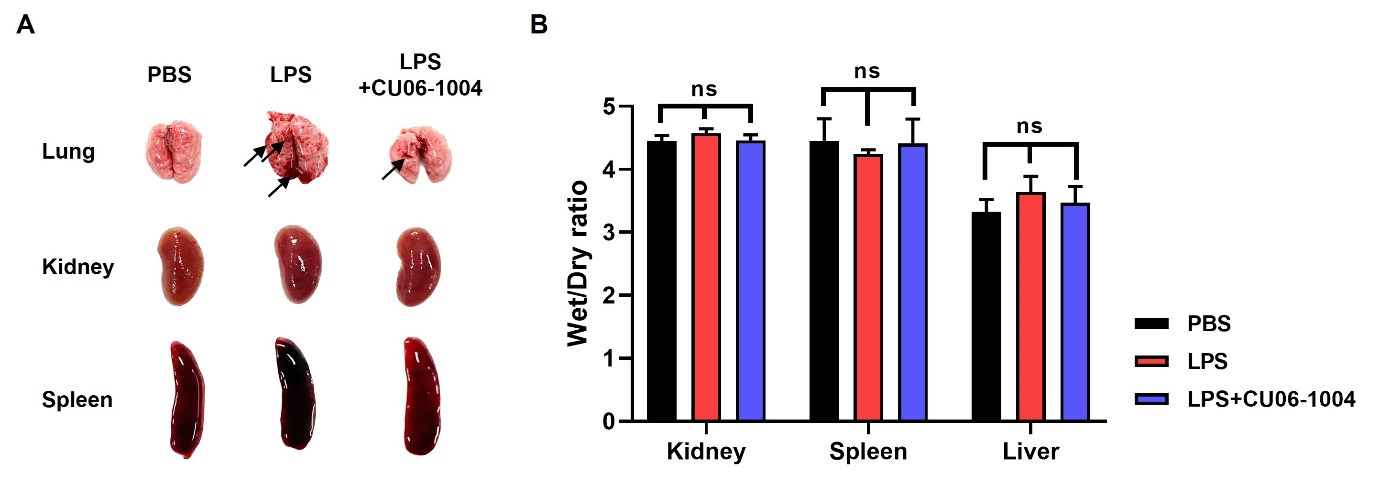
**

**Supplementary Figure 1. CU06-1004 reduced attachment of immune cells on pulmonary vascular lumen.** SEM images of lung tissues from ALI mouse model. Three magnifications (200X; first row, 500X; middle row, and 1,000X; last row) were shown. Sectioned pulmonary vessels of each group were presented. Scale bar of 1,000X magnification: 10 µm.

**Supplementary Figure 2. CU06-1004 restored endothelial cell-to-cell contacts and swollen morphology after LPS challenge.** Representative images of pulmonary endothelium from mouse lung of LPS challenged using TEM. Low magnification (20,000X, upper panel) and high magnification (60,000X, lower panel) were shown. Endothelial cell-to-cell contact (red arrow) was presented in the upper panel, and swollen morphology (two-headed arrow) was presented in the lower panel. Scale bar of 60,000X magnification: 500 nm.

**Supplementary Figure 3. Visual appearance and edema of tissues in mouse. (A)** Representative images of lung, kidney, and spleen after LPS challenge. Arrows indicated hemorrhage and congestion regions. (B) Edema of tissues (kidney, spleen, and liver) was described as wet/dry ratio. N=3 per group. ns: not significant.
