# Supplementary figures and images for: Efficacy of CU06-1004 via regulation of inflammation and endothelial permeability in LPS-induced acute lung injury
Source: J Inflamm (Lond). 2023 Apr 6;20:13. doi: 10.1186/s12950-023-00338-x (PMC10078077; doi:10.1186/s12950-023-00338-x)

**Additional file 2. Original blot images of Figure 6B**


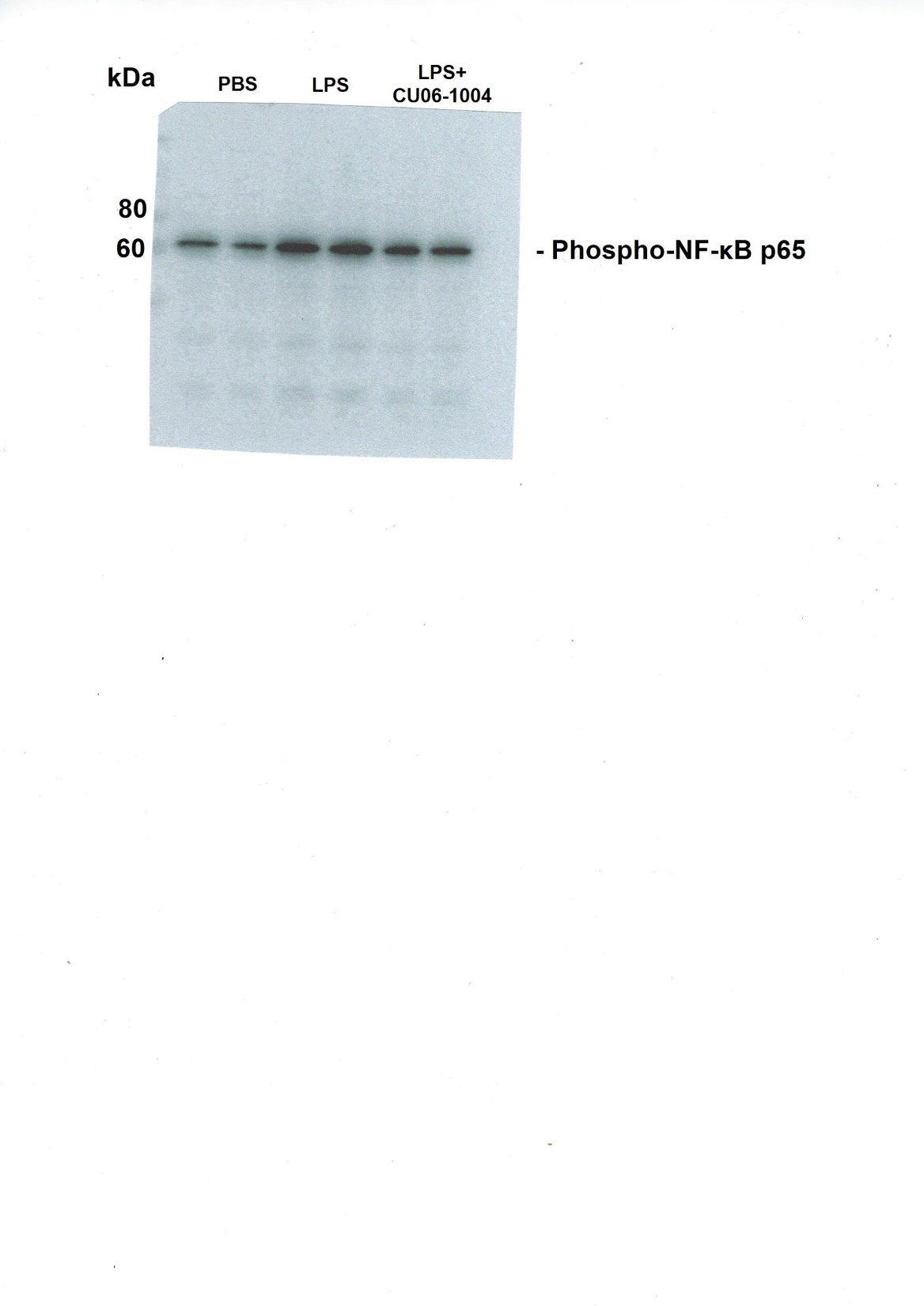


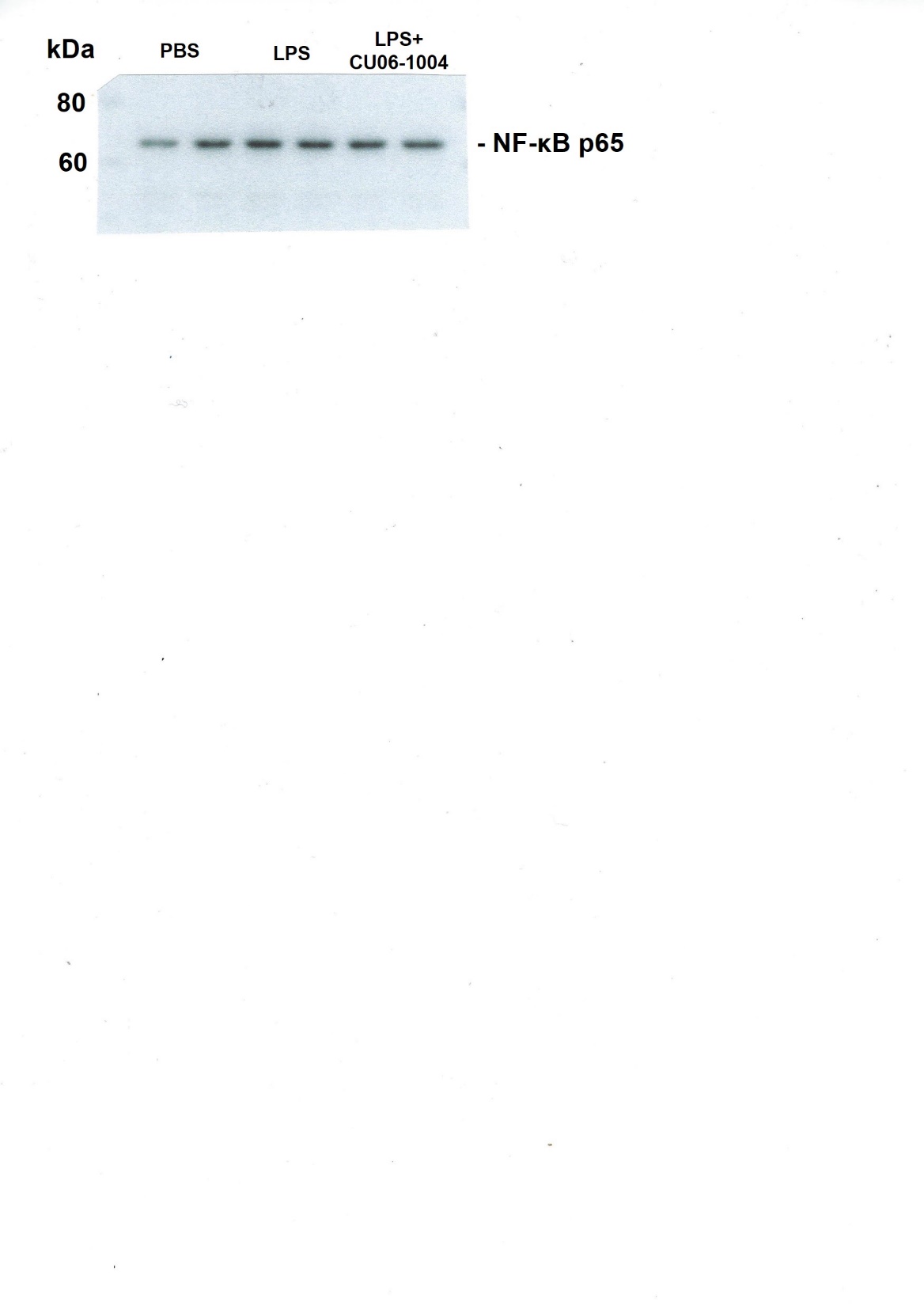


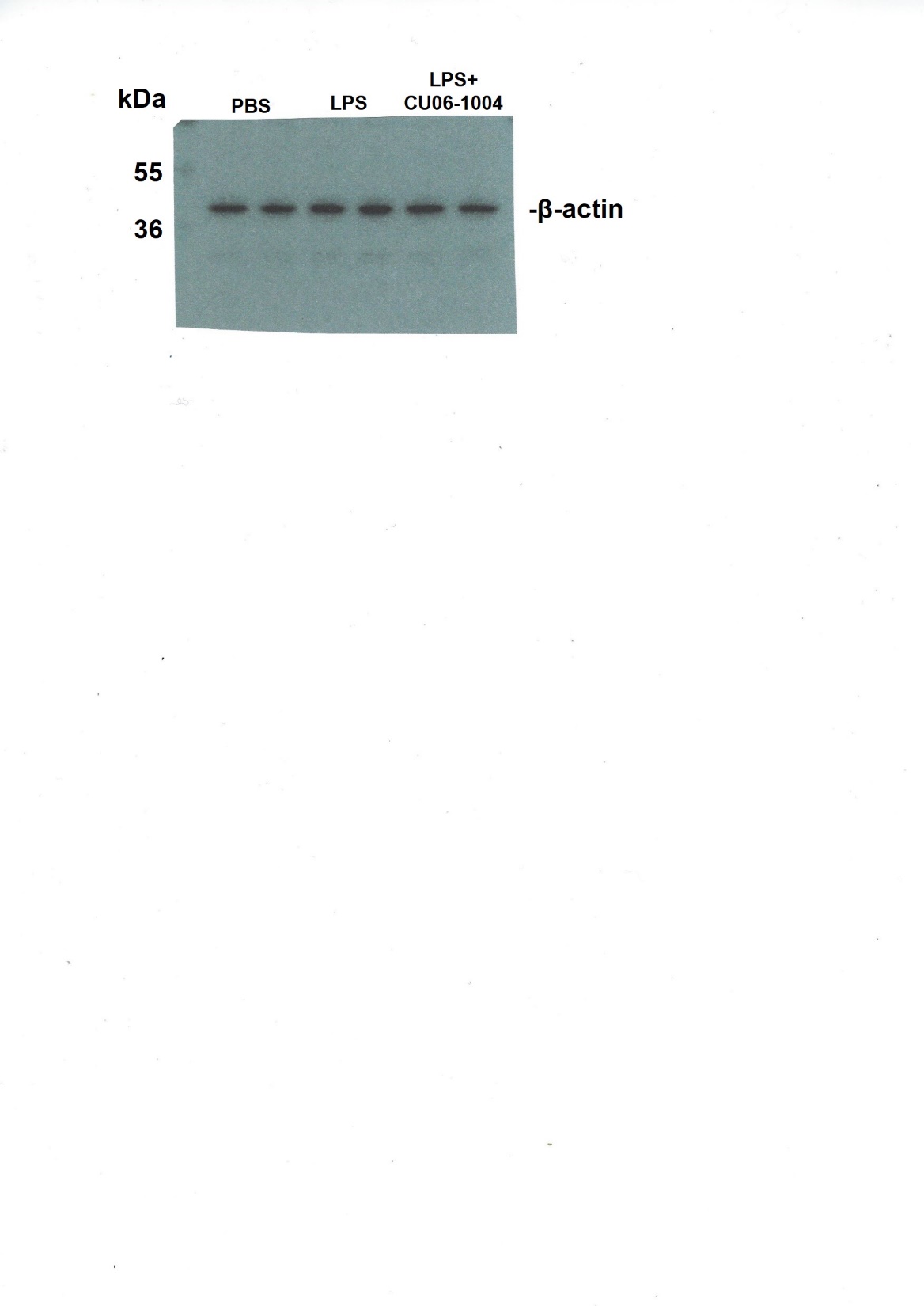

Supplement: Supplementary file 2 — Additional file 2: Original blot images of Figure 6B [file 12950_2023_338_MOESM2_ESM.docx]
